# Supplementary material for: Oviduct Transcriptomic Reveals the Regulation of mRNAs and lncRNAs Related to Goat Prolificacy in the Luteal Phase
Source: Animals (Basel). 2022 Oct 18;12(20):2823. doi: 10.3390/ani12202823 (PMC9597788; doi:10.3390/ani12202823)
Supplement: Supplementary file 1 [file animals-12-02823-s001.zip › Supplementary figures.pdf]

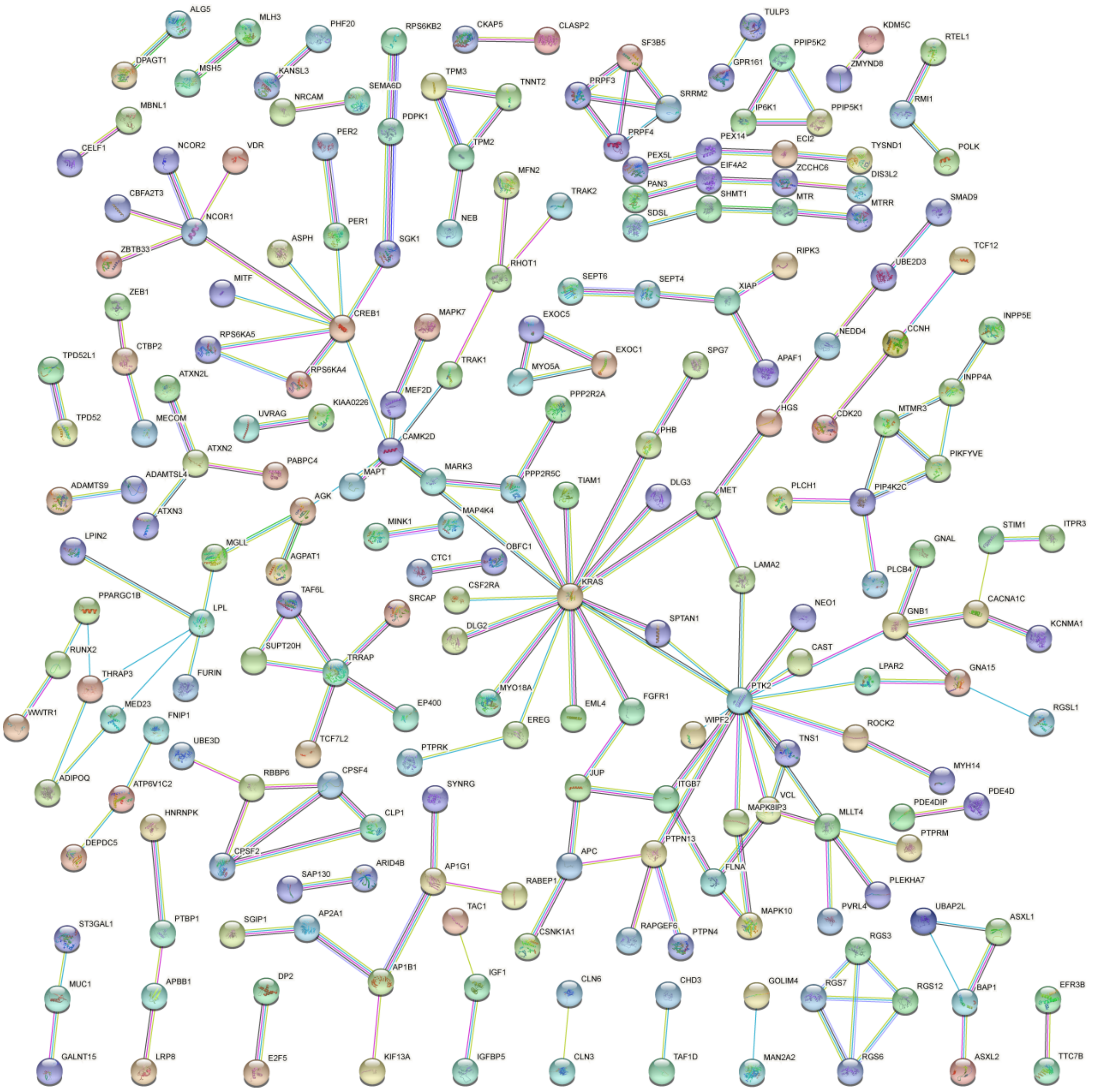

Figure S1. PPI network was constructed by Search Tool for the Retrieval of Interacting Genes/Proteins of the DE-mRNA-coding protein in LL vs. LH. The minimum required interaction score was set as 0.9.

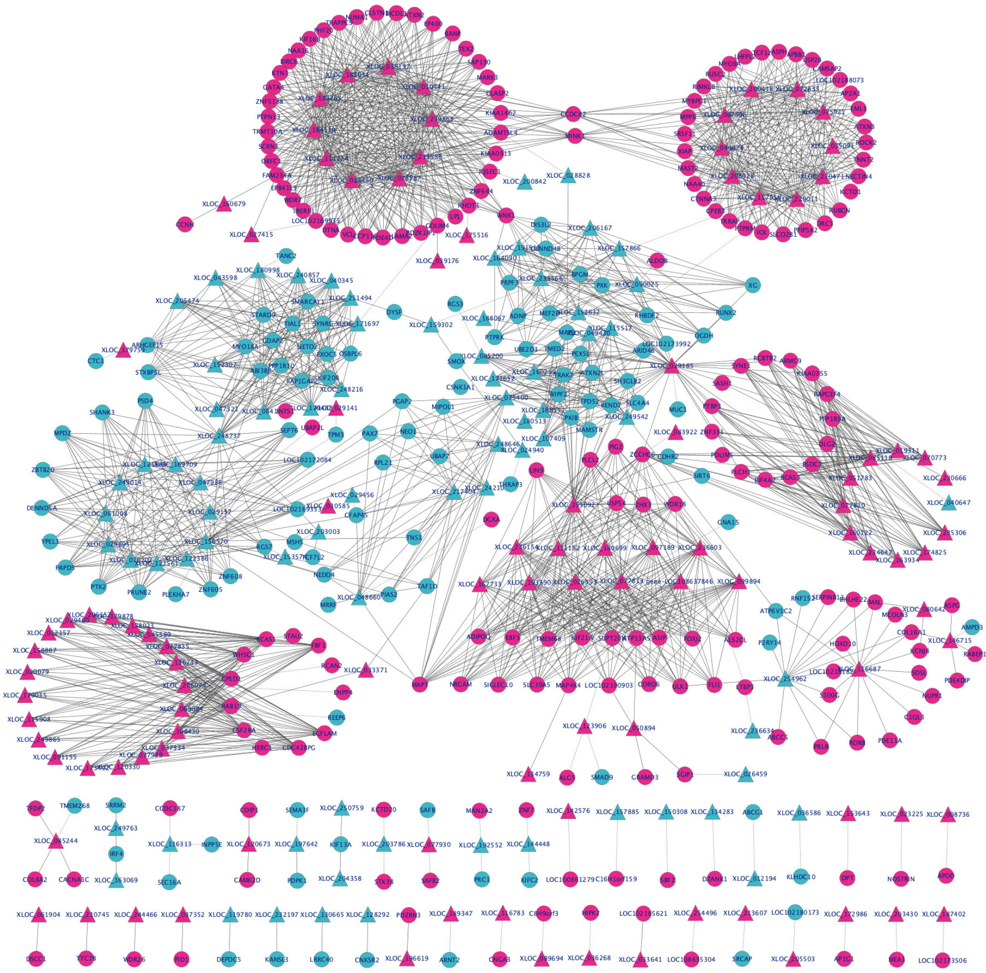

Figure S2. Interactions of the differentially expressed lncRNAs (DELs) with target genes form a network.
